# The Role of Oral Fusobacterium nucleatum in Female Breast Cancer: A Systematic Review and Meta-Analysis

**DOI:** 10.1155/2022/1876275

**Published:** 2022-11-23

**Authors:** Fariah I. Gaba, Raquel Carcelén González, Raquel González Martïnez

**Affiliations:** ^1^Mondzorg Scheveningen, Renbaanstraat 75, 2586 EZ, The Hague, Netherlands; ^2^Faculty of Health and Science, CEU Cardenal Herrera University, Carrer Lluís Vives 1, 46115 Alfara del Patriarca, Valencia, Spain; ^3^CIMEV Institute in Spain, Periodontics and Oral Surgery at the Faculty of Health and Science, CEU Cardenal Herrera University, Carrer Lluís Vives 1, 46115 Alfara del Patriarca, Valencia, Spain

## Abstract

**Introduction:**

Breast cancer is the world's most prevalent malignancy, with an increasing incidence and a predisposition for postpubertal females from all cultural and ethnic backgrounds. More recently, oral Fusobacterium nucleatum species have been observed in cancerous human breast tissue, drawing attention to the role of microbes in cancer pathogenesis.

**Objectives:**

Investigating oral Fusobacterium nucleatum species as potential biomarkers for female-specific breast cancer.

**Methods:**

A systematic search in The Central Register of Controlled Trials, EMBASE, EBSCO, NCBI, and MEDLINE databases was undertaken from the 1^st^ January, 1983–31^st^ March, 2022. Articles included were in English and based on women between the ages of 18–96 years with confirmed gingivitis/periodontal disease and breast cancer diagnoses from registered specialists. Authors extracted data independently, and a meta-analysis of risk estimations measuring associations between oral Fusobacterium nucleatum species and female-specific breast cancer was elucidated via calculated relative risks and 95% confidence intervals.

**Results:**

AXIS tool analysis revealed 78.70% of articles with a positive correlation between oral Fusobacterium nucleatum and female-specific breast cancer. The risk of breast cancer development increased with significant levels of oral Fusobacterium nucleatum due to gingivitis/periodontitis (relative risk = 1.78, 95% confidence interval = 1.63–1.91). Low-moderate statistical heterogeneity was found (*I*^2^ = 41.39%; *P* = 0.02), and the importance of periodontal status on breast cancer pathogenesis was determined (relative risk = 1.24, 95% confidence interval = 1.01–1.30).

**Conclusions:**

Oral Fusobacterium nucleatum species are a risk factor for breast cancer development, thus elevating their biomarker potentiality.

## 1. Introduction

Breast cancer (BC) is the world's most prevalent malignancy, with a predisposition for postpubertal females [1]. In 2020, 2. 261, 419 million women were diagnosed with BC, resulting in 684,996 deaths worldwide [2]. Estimations suggest that 1 in 8 women will be diagnosed with pernicious BCs during their lifetime [3]. Numerous treatments are available, such as radio- and chemotherapies, anticancer medicaments, hormone therapy, and strategically targeted biological therapies. Over the past decade, mortality rates have significantly diminished, with elevations in BC survival rates [1].

Despite all international efforts to raise public awareness, outreach programmes, frequent testing for the condition in women above 40 years, and extensive research into the various cell-signalling pathways responsible for BC development, its incidence is rising amongst postpubertal females of all cultural and ethnic backgrounds [1–3].

Attention has been drawn to the significance of microbes and their role in cancer pathology initiation/activation, with further support for an association between disruptive microbial homeostasis and BC [4–8]. The oral cavity is a cornucopia of microorganisms, with approximately 700 identifiable bacterial species that collectively encompass the oral microbiome [4]. Its environment contains particular oral habitats, such as the hard, nonshedding external surface of human dentition, the gingiva, and the dorsum of the tongue. These appreciable loci permit unique microbial niche formations creating long-short distance microbial interactions [4].

When the intraoral environment becomes overburdened with dental plaque accumulations, this can cause immediate microbial dysbiosis, initiating acute inflammatory responses that result in gingivitis [9]. Accumulation of dental plaque over prolonged periods instigates chronic inflammatory responses, causing periodontitis [9]. Periodontitis is a rife disease associated with both microbial dysbiosis and inflammatory responses, affecting approximately 45–50% of adults >30 years old and >60% of people aged >60 years old [10]. It is characterised by loss of connective tissue attachment, gingival inflammation, alveolar bone resorption, and loss of dentition [10]. Disparate from other infectious diseases, periodontitis is caused by bacteria from within the oral cavity and accelerated by an increase in microbial biomass as opposed to compositional alterations within a specific bacterial community. This premise concurs with an apparent increase in bacterial load (and so periodontal disease) in murine models and in humans as they grow older [11]. During the progression of inflammation in PD, a multifunctional molecule (endothelin (ET)-1) is overexpressed within the human body, secreted by endothelial cells after exposure to pathogenic bacteria, and is a dominant vascular inflammatory mediator [10, 12]. Reports have stipulated that serum proinflammatory cytokines such as IL–1, IL-6, and IL-8 are responsible for the upregulation of ET-113 [10, 13]. Furthermore, the conjecture that a combination of chronic inflammatory markers associated with systemic diseases, certain oral bacteria and their by-products leaving the oral cavity and infiltrating the systemic circulatory system, aids cancer progression to distant bodily sites [14] Fusobacterium nucleatum (FN) is one such important virulent pathogen. It is a spindle-shaped, nonspore-forming anaerobe, identified as one of the most common gram-negative oral microbial species [15, 16], with a significant contribution to biofilm development and maturation accompanying dysbiotic alterations in dental plaque [9]. More recently, FN has been observed in cancerous human breast tissue, with stimulation of BC advancement in murine models. [17, 18], and a plethora of articles have been published over the past decade evidencing a link between the microbiome and cancer [19–21]. In 2020, a study of breast ductal carcinoma cells in situ (BDCIS) were exposed to have significantly elevated immunoreactivity for ET-1 when compared to healthy breast tissue. Furthermore, significant levels of immunoreactivity were demonstrable in invasive tumours when contrasted with BDCIS [22].

The results highlight the actively prominent role played by oral FN species and endothelin molecules in the creation of a perfect proinflammatory microenvironment evident in individuals diagnosed with PD and its link to accelerating BC pathogenesis [23–25].

Over time, innovative periodontal surgical techniques have come into play with the purpose of slowing/arresting PD progression and regenerating lost periodontal tissues. Several flap surgical designs have been proposed with modifications to minimise periodontal surgical trauma (PST); however, the traditional method to access flap surgery (2 flap reflections of buccal and lingual/palatal) remains the advocated surgical procedure for pocket reduction/elimination [26]. This is where minimally invasive surgery (MIS) has been introduced to appease such issues [27].

MIS is a novel approach that aims to respect hard and soft tissues via minimal flap reflections, thus reducing postoperative pain, encouraging healing, and improving clinical outcomes [28]. Since 2007, the single flap approach (SFA) has been modified and evolved to better suit a more minimally invasive surgical technique (MIST) for PD osseous defects, with the purpose in minimising surgical trauma. A 2013 study reported minimal bone loss when utilising MIST compared with conventional surgeries [29]. SFA permits flap repositioning and attachment to the undetached papilla, thereby inhibiting contamination by blood clots and reducing post–operative recession [30]. This reduces the risk of stimulating PD bacteria by upregulating proinflammatory cytokines and endothelins, which enter the systemic circulatory system and travel to distant anatomical sites, potentially instigating BC pathogenesis. Moreover, performing MIST using dental loupes increases precision, enhances rapid wound healing, and reduces postoperative pain [30].

The significance and functionality of the human microbiome are mainly derived from a plethora of studies into the human gut, the origin of approximately 99% of the microbial mass responsible for the maintenance of homeostatic gross metabolic function [31]. Comparatively, there are far fewer articles evaluating the importance of the oral microbiome and its role in breast cancer pathogenesis. To our knowledge, no meta-analysis has been conducted specifically on FN species, examining its potential as a biomarker for female-specific BC. With this in mind, our aims are to: (i) highlight the evidence to date and understand the impact of oral FN species and their link in BC pathogenesis; (ii) examine the link between endothelin (ET-1) molecules and oral FN species and their roles in inflammatory cytokine activation, PD, and BC pathogenesis; (iii) discuss surgical treatments for PD (specifically periodontal flap designs) and the associated increase in risk of PD onset; (iv) appreciate the fundamental part of oral FN species in BC pathogenesis and their potentiality as BC biomarkers (v) raising awareness of the complex interactions of oral bacteria and inflammatory cytokines at a cellular level as a novel approach for therapeutic targets, thus improving future PD and BC prognosis.

## 2. Methods

### 2.1. Protocols and Registration

This systematic review was created in accordance with the preferred reporting system for systematic reviews and meta-analyses (PRISMA) [32]. Also refer to the PRISMA 2020 expanded checklist in the supplemental appendix: Description 1. FG, RCG, and RGM carried out thorough, centralised evaluations of peer-reviewed publications on oral FN and BC pathogenesis.

### 2.2. Search Strategy

The P.I.C.O.S model of clinical questioning for evidence-based medicine was implemented by FG, RCG, and RGM as the defining criteria for article incorporation, utilising the P.I.C.O.S point system (Supplemental Appendix: Table 1), to create a summarisation of included articles analysing oral FN and BC pathogenesis (Supplemental Appendix: Table 2). A MeSH search string of studies involving the following key words was included: oral microbiome, Fusobacterium nucleatum, breast cancer, neoplasms, and adult women (Supplemental Appendix: Table 3).

### 2.3. Article Selection

The objective of this systematic review was to examine the importance of oral FN species and their potential role as biomarkers for female-specific BC. Women younger than 18 years of age were excluded. Full-text articles published from January 1, 1983, up to and including 31^st^ March, 2022 involving human, postpubertal females were included for analysis. Refer to Table 1for a detailed description of the inclusion and exclusion criteria.

### 2.4. Search Protocol

A variety of databases were thoroughly searched as recommended by COCHRANE (The Central Register of Controlled Trials), EMBASE, EBSCO, NCBI, and MEDLINE. The time period from which databases were searched by FG, RCG, and RGM was from February 1^st^, 2022–February 25^th^, 2022. The abstracts and titles of the articles recovered in the search were appraised within the context of the study criteria, and nonrelevant articles were discarded. Full-text articles were screened with respect to the inclusion and exclusion criteria (Table 1).

In total, three separate searches were conducted and categorised into 3 phases: phase 1 was the initial search undertaken by FG, RCG, and RGM with the purpose of collating and evaluating articles linking human microbiome dysbiosis to cancer development. Phase 2, undertaken by FG and RCG, was a more “concise” search of articles focussing on a dysbiotic relationship between varying ecosystems belonging only to the human oral microbiome and cancer development. The final stage, supervised by FIG (phase 3), focussed on articles investigating a link between oral FN bacterial species and their influence on female-specific BC pathogenesis.

In all 3 phases, abstracts and titles were independently reviewed by FG and RCG. FG collated the required data from the selected articles to be incorporated, and data extraction were cross-checked by FG, RCG, and RGM. In instances where author discrepancy was discovered in phase 1, articles were reanalysed and progressed to phase 2. If discrepancies remained, a consensus was reached by requesting involvement by the co-authors, with FG having the final say.

### 2.5. Quality Assessment Measures

**Figure 1:**
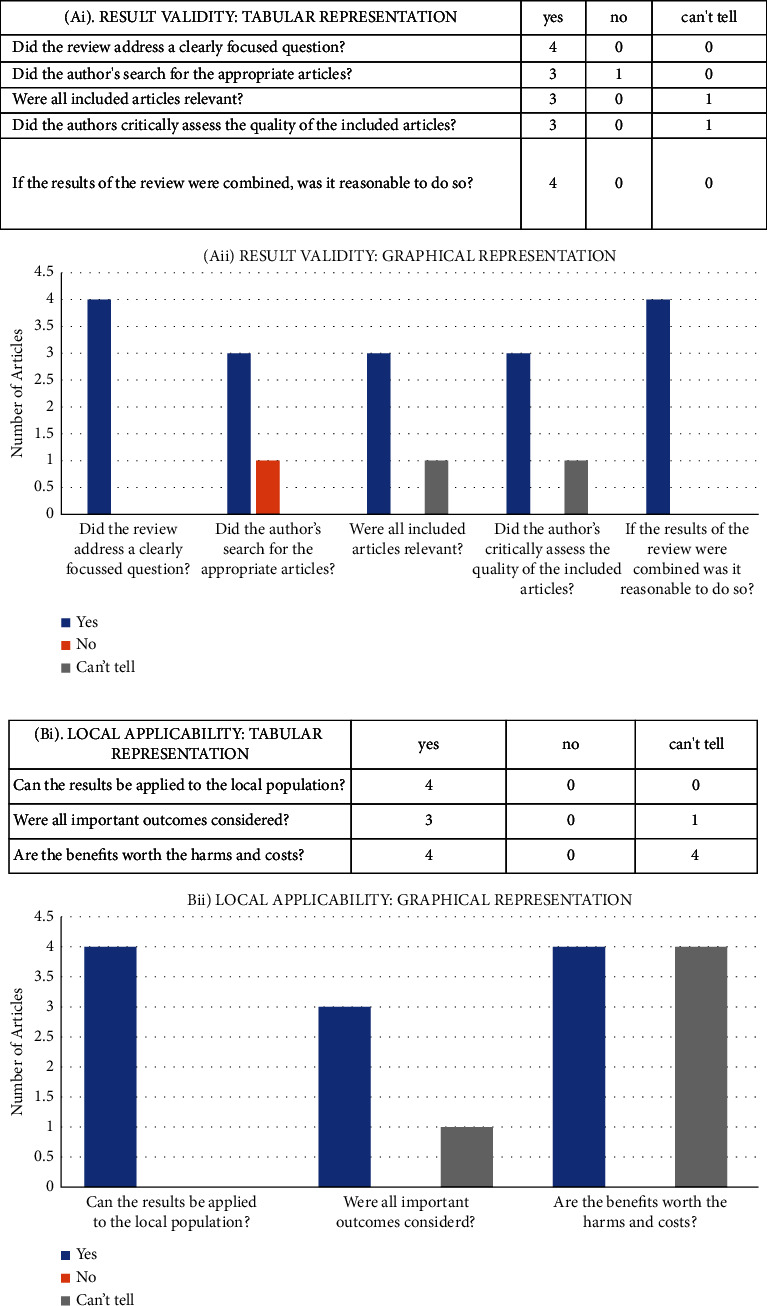
Summary of critical appraisal skills programme tool (CASP) for the evaluation of literature reviews with respect to (a) result validity and (b) local applicability.

The methodological sections of 6 out of the 10 articles were analysed by FG, RCG, and RGM utilising the AXIS Tool (Supplemental Appendix: Figure 1), which consists of a fixed set of questions intended to scrutinize data acquisition procedures as stated in the articles while considering a myriad of factors such as the adequacy in representation of participant population, inclusion of appropriate population sample sizes, consideration of nonresponding participants, and ultimately the effect of the acquired statistical data on study reliability and consequent effect on study outcomes [35]. The results are illustrated in the supplemental appendix section: Figure 1. The Critical Appraisal Skills Programme (CASP) Tool was utilised by FG and RCG to appraise 4 of the included articles, questioning the obtained and stated result validity of studies and to assessing the local applicability of result findings thus enabling extrapolation to the wider population [36]. These results are depicted in Figure 1.

### 2.6. Outcome Measures and Investigated Methodologies for Oral FN Measurements

The primary evaluated outcome is to establish a link between oral FN species and BC pathogenesis in adult women. Articles examining BC pervasiveness and its association with oral microbiome imbalance (with reference to FN) were considered for inclusion. Our focus for this systematic review was on cancer development and not on survival/mortality rates.

To examine specific bacterial communities, DNA microarrays create high-throughput, quantitative and systematic analysis—especially when examining oral microbiota [37]. However, other culture-independent“omic”-based methodologies, such as gel-based and polymerase chain reactive (PCR) techniques, were included.

### 2.7. Statistical Analysis

**Figure 2:**
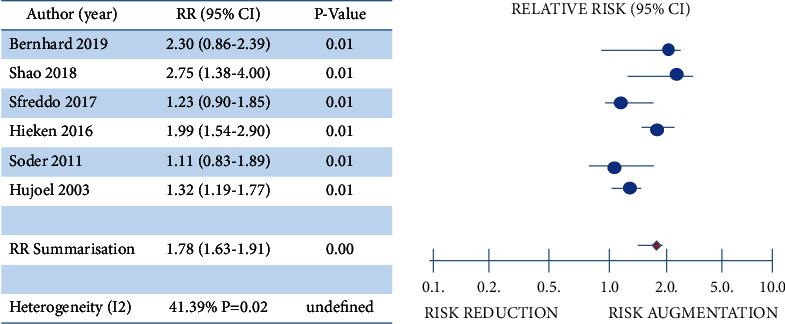
Summarisation of the overall results of articles included in AXIS tool analysis.

From the 10 articles included, 6 articles underwent AXIS Tool and meta-critical analysis [38–43] and 4 underwent CASP Tool analysis [4, 18, 44, 45]. Calculations and estimates of the measures of association between oral FN species and female-specific BC were calculated from the extracted data. Microsoft Excel was utilised for graphical data representation. The results of the quality assessment of included articles were presented in tables or figures (Figures 1, 2 and Table 2. Supplemental Appendix: Figure 1).

## 3. Results

### 3.1. Search Results and Geographical Spread of Investigated Populations

Initially, 238 articles, including duplicates, were retrieved. 164 studies were excluded (68.91%), and following duplicate eliminations (*n* = 49), 189 relevant articles were included (79.41%).

**Figure 3:**
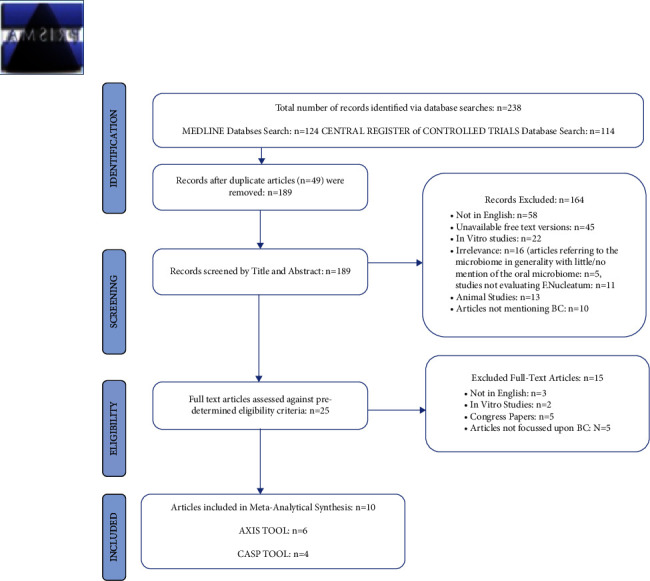
The PRISMA flow diagram outlines the search and selection process applied in preparation for this review.

25 articles were eligible for inclusion (13.23%), with a final total of 10 studies eventually included for analysis (40.0%), consisting of 1745 subjects (Figure 3). Articles were excluded if: (a) studies were not written in English (b) studies were irrelevant (c) study results were generated using “in vitro” methodologies (d) free-text versions of articles were unavailable (e) articles generated results based solely upon animal experimentation (f) studies did not mention/investigate a link between FN and BC.

Our results revealed that 5 studies were implemented in North America (*n* samples = 45,622, *n* patients = 1348), 3 in South America (*n* samples = 1056, *n* patients = 331), 2 Europe (*n* samples = 1586, n patients = 66). The female population age range was 18–96 years, with a generalised trend of case participants being older than healthy female control participants. An extended range of articles were sourced, with a range of publication dates from 1983–2022.

### 3.2. General Analysis

An AXIS tool analysis of 6 of the included articles [38–43] revealed that 78.70% of articles found a positive correlation [39, 41, 42], 6.48% found no correlation [40], and 12.96% of articles [38, 43] were inconclusive with respect to the ascertainment of a link between the presence of oral FN species and BC in adult women (Supplemental Appendix: Figure 1).

Graphical illustrations generated via CASP Tool analysis of the other 4 included articles [4, 18, 44, 45] clearly demonstrate result validity with respect to the author's manner of critical data assessment, search methodology for the inclusion of relevant data, and the proportion of relevant articles included (Figure 1(a)). Our investigation into the overall applicability of local results was positive, indicating that the authors of more than half of the included articles considered all outcomes important (Figure 1(b)).

### 3.3. Metacritic Analysis

A more detailed evaluation of the data provided by the 6 included articles was implemented, and details of article study design, follow-up periods, and dental status were examined (Table 2). How article study design influenced the final result outcomes, was scrutinised via stratification analyses as conceded by retrospective cohort study design (*n* = 1) [42], case-control study design (*n* = 1) [41], and prospective cohorts (*n* = 4) [38–40, 43].

The findings highlighted a statistical difference between the included studies (prospective cohort: RR = 1.18, 95% CI = 0.95–1.40; retrospective cohort: RR = 1.22, 95% CI = 1.11–1.39; case control: RR = 2.10, 95% CI = 1.15–2.78) (Table 2). Furthermore, our results insinuate a 1.78 risk augmentation of BC development in those women with significant levels of oral FN presence due to poor dental hygiene resulting in gingivitis in the short term or periodontitis in the long term (RR = 1.78, 95% CI = 1.63–1.91; Figure 2). Low-moderate statistical heterogeneity was apparent across all 6 articles (*I*^2^ = 41.39%; *P* = 0.02; Figure 2).

With respect to the evaluation of specified follow-up durational data from the included articles, it was apparent that 2 studies [38, 39], which stated their patient follow-up durations as being for over a period of less than 10 years, concurred to the significant impact that FN species within oral biofilm has on the promotion of female-specific BC pathogenesis (RR = 1.18, 95% CI = 1.15–1.29) (Table 2). Comparatively, 2 of the included studies [41, 43], both of which stated their patient follow-up periods as being more than 10 years, implied that the influence of FN species microbial levels within oral biofilms statistically possessed an insignificant effect on female-specific BC pathogenesis (RR = 1.41, 95% CI = 0.88–1.87) (Table 2). Evaluation of all 6 included articles [38–43] revealed the importance of periodontal status (implying the negative effects of oral FN microbial dysbiosis) on BC pathogenesis (RR = 1.24, 95% CI = 1.01–1.30) (Table 2).

## 4. Discussion

The result of this systematic review reveals a link between oral FN species and their role in female-specific BC pathology, thus elevating their potential to act as biomarkers in BC. However, from our subgroup calculations, it can be elucidated that numerous cofounders, such as follow-up periods and study designs, may have influenced the overall evaluations.

Over the past decade, much research effort has been instigated with particular emphasis to the understanding of oral microbial burden impaction on an individual's overall systemic health, and numerous articles [46–48] have alluded to a connection between a state of chronic gingival inflammation and BC development, of which oral FN species have been named as a significant contributor [38, 39].

The Human Oral Microbiome Database (HOMD) was conceived and setup in 2005, providing vital information on the types of bacterial niches present within the oral cavity [45]. Within a recent period, 1,576 genomes and 784 different taxa within the oral cavity have been identified and subsequently listed [49], of which FN species were representative of one of the most prevalent phyla within the adult oral cavity [50–53].

Although the establishment of polymicrobial films as essential participants in the initiation and progression of periodontitis has been validated, some questions remain unanswered—the most important being how microbial dysbiosis and fluctuating microbial compositions within the oral cavity are able to instigate periodontal pathogenesis and if specific microbial characteristics relate to other systemic diseases. More recently, the emergence of meta-transcriptomics and metagenomics has permitted high-throughput analysis of microbial communities, thus illuminating some aspects of this conundrum.

Traditionally, microbial detection and identification involved the implementation of culture methodologies, including sample preparations, plate counts, and the isolation of singular bacterial colonies [54]. However, metagenomics is different in that it genomically analyses all microbial communities, evaluating functional relationships, population structure and diversities, and the interactions of these microbial communities within their specific environments [55]. The evident advantages of metagenomics are not only restricted to its ability to manufacture significant quantities of sequencing data approximately between 120 giga-bases–1.5 tera-bases/run [56], it has the capacity to interpret the functional diversities of microbial populations and discover different species/genes with certain functionalities [54]. More recently, an increasing number of articles have postulated that periodontal pathogens are a causative link to the development and aid progression of certain systemic diseases, including cancer [57]. Despite the technology's positives, there are major shortcomings in the methodologies of several of the current studies investigating the efficacy of metagenomics, such as (i) inadequate sample sizes, thus affecting the efficacy of periodontal treatment on regulating periodontal microbial populations, (ii) study designs, (iii) sampling methods, and (iv) follow -up durations are all variable questioning its effectiveness in periodontal treatment evaluations [58].

In 2022, a review article examined the possible mechanisms by which oral microbial FN species were able to colonise the lactiferous ducts of breast tissue, via translocation into the systemic circulatory system [44]. A further study revealed that oral FN species were able to colonise cancerous tumoral growths via specific attachment to Gal-GalNAc receptors displayed on the surface membranes of tumour cells [59], implying that oral FN species can reach other Gal-GalNAc–displaying tumours via the same mechanism. Therefore, as haematogenous Gal-GalNAc levels augment, female-specific BC progression also accelerates [44].

Fap2 of FN species is a galactose-inhibitable adhesin involved in coaggregation and cell adhesion [18]. Results acquired “in vivo” from experimentations implemented on murine models revealed that FN-induced BC tumoral growths and their metastatic advancements were Fap2-dependant and reversible with antibiotics [18]. A further study in 2019 involved the evaluation of the microbial abundances of over 40 bacterial species within 144 subgingival plaque samples [38]. One of the highest mean counts of infectious pathogens obtained from the samples included that of the FN species, thus drawing a strong association between oral FN species-induced chronic inflammation as one of the risk factors for female BC [38].

A comparative study in 2016 undertook extensive analysis of microbial communities originating from female breast tissue samples of both benign and malignant growths. The findings deduced that there was a direct correlation between the microbial enrichment of FN species and that of female-specific BC malignancies [39]. The inference from these results implies that oral FN species have significant potential as biomarkers for BC affecting adult females, and by specifically targeting oral FN species, it could significantly improve BC prognosis.

Contradictorily, a study in 2003 evaluating data from 11, 328 participants with a follow-up period between 11 and 21 years, including the NHANES I follow-up within the aforementioned time period, demonstrated no relationship between gingivitis or periodontitis and BC diagnosis in adult women [40]. A further study conducted in 2016 discovered no direct association between periodontal pathogens present within the oral cavity of the included participants and total/site-specific cancer incidences—including BC. (302) These opposing findings can be explained and could be a direct consequence of the following: (i) distinctiveness of the study design, (ii) whether periodontal assessment was factored into individual article assessments by the authors, (iii) author(s) adjustment for confounders, (iv) variable patient follow-up periods, and (v) patient sample sizes [41, 42]. Nonetheless, the relationship between oral microbiota in its entirety and the possible utilisation of specific microbial species in detecting and diagnosing female-specific BC remains controversial.

An imperative aspect to consider is the importance of PD surgical procedures, the influence of “flap designs” and utilisation of dental loupes on the risk of developing periodontitis. Conventionally, DFAs are the primary procedures implemented for direct root access and pocket elimination. Nonetheless, there are innumerable disadvantages such as membrane exposure, flap dehiscence, oedema, the absence of primary closure of interdental spaces, and bleeding [60]. With increasing importance being placed on SFA MIST and concomitant use of dental loupes, numerous articles concur that clinical outcomes of delicate tissues such as the gingiva are greatly improved as a result due to (i) reduced bleeding during operative procedures, (ii) minimal flap reflections without periosteal incisions, and (iii) precise incisions promoting healing by primary intention via easy stabilisation of the undetached papilla [30, 61–63].

Preservation of intact papilla via MIST SFA promotes wound stabilisation permitting uneventful tissue formation and maturation [64].

### 4.1. Review Strengths

Key strengths of this review included the spectrum of databases searched and the large number of included articles, thereby strengthening our statistical analysis. Additionally, all included articles and their reference lists were thoroughly examined by the authors, and cross-matched against the predetermined inclusion and exclusion criteria, thus enhancing the reliability of this systematic review. Furthermore, the use of the rigorous evaluator “AXIS Tool” for the scrutinization of information presented in primary research articles permitted more in-depth assessment of individual aspects of study design, providing a complete and overall qualitative analysis. This subjectivity permitted greater flexibility by incorporating quality of reporting concomitantly with risk of bias when assessing articles and providing a clear advantage over other appraisal tools such as the “Cochrane Risk of Bias Tool” which fails to address poor quality reportage [65].

### 4.2. Review Limitations

Despite our best attempts, some review limitations have been identified. For example, one included study within the meta-analysis was a definitive contributor of result heterogeneity [43]. However, the exact reasons were unable to be precisely identified. Additionally, there is a strong possibility that patient control selection bias was inadvertently introduced via the deliberate recruitment of individuals without gum disease to represent control groups in the included articles. There were evident discrepancies in definitions of PD that varied between the articles examined, with some utilising various defining means to confirm PD diagnosis such as self-reported disease status, tooth mobility, and patient medical records, thus introducing article heterogeneity. Lastly, the relationship between oral FN species and their involvement in either the instigation/development of tumour stages in women were unable to be considered because of limitations of data availability.

### 4.3. Future Prospects

Our search focussed on an array of observational and primary studies, literature reviews, and comparative and case studies. Despite our investigation having revealed a link between oral FN species and female-specific BC, to date there is still deficits of current data availability.

With this in mind, investigations into this field with more focussed assessment objectives would be useful, thus taking the following into consideration: (i) IIF-“Identification-Isolation- Flag-up” of sub-types of FN species presence within the varying sections of breast tissue from a population of women with confirmed BC at different disease developmental stages (lowest grade-highest grade). This would allow for a precise analysis of microbial population counts and examination of the proportional activity levels of each subtype of FN species present. (ii) The exact same procedure would be implemented in a population of women with confirmed diagnoses of varying oral inflammatory conditions and at varying stages: (A) gingivitis (mild-moderate-severe) and (B) periodontitis (mild-moderate-severe).

We strongly believe that multiomics analysis [66] has immense potential for our proposal with respect to the discernment of complex relationships between the human microbiome and cancer risk. We suggest that integration of microbiome data with data obtained from other omics data of interest would permit understanding of causal factors for cancer development. Currently, microbiome multiomics data analysis is still challenging due to deficient tool availability [67]. To remediate this, further well-designed RCTs with lengthened follow-up periods would help confirm whether meta-transcriptomic/meta-genomic alterations are viable contributors to personalised periodontal therapies.

Our motion would aid DHCP's in the promotion of good oral hygiene maintenance (especially in adult women, as there has been previous mention of its necessity in a prior study) [42] and could revolutionise the field of dentistry by promoting tailored treatments of high-risk patients/patients suffering from varying degrees of gingivitis and/or periodontitis, against the translocation of specific oral FN species into the systemic system.

## 5. Conclusion

This systematic review and meta-analysis imply a significant relationship between oral FN species and their role in female-specific BC pathogenesis, highlighting their biomarker potentiality. Our revelations raise awareness of the intricacies of this relationship with respect to adult female populations and could potentially help to reduce/prevent the risk of BC development worldwide, fundamentally transforming the future of both medical and dental prognostics.

## Figures and Tables

**Table 1:** Inclusion and exclusion criteria. RCT = Randomised controlled trials. (a) In instances where numerous studies examined the same study populations, the longest period of follow-ups were selectively extracted and interpreted; (b) with satisfactory data provisions permitting data and methodology comparability between included articles. (c) Each area of the tooth is scored from 0 to3 (mesial, distal, vestibular, palatine, or lingual). The GI score per tooth is the sum of the scores from the four areas, divided by four. The GI for the subject = Sum of the indices of each tooth divided by the number of teeth examined.

Classification	Criteria	
	Inclusion	Exclusion
Publication dates:	Articles published from 01/01/83–31/03/22	Publications >30 years old;
Language:	Articles in English	Articles unavailable in English
Study population	Adult women with breast cancer, diagnosed by certified and registered gynecologists specialized in mastology	Women requiring antibiotic prophylaxis prior to oral examinations
		Women with diagnostically confirmed BC malignancies.
	Women between the ages of 18–96 years	Adult females undergoing orthodontic treatment and thus wearing fixed orthodontic
	Participants evaluated by a fully registered and qualified dentist and who after clinical, histopathological and radiographic evaluations, are currently demonstrating (i) gingivitis	Appliances
	Clinical presentation: Utilisation of the GI grading system c	The usage of medications with inadvertent side effects resulting in gingival hyperplasia-nifedipine, cyclosporine, phenytoin
	Grade I: Mild inflammation–a slight color change, slight oedema confined to the interdental papillary region. No bleeding on probing	Females diagnosed with psychomotor disorders and who had taken antibiotics within 6 months before clinical examination.
	Grade II: Moderate inflammation–redness, oedema with involvement of both marginal gingiva and the interdental papilla, and glazing. Bleeding on probing	Pregnant females
		Females taking oral contraceptives
		Females diagnosed with Vitamin C deficiency and those with a “high refined–carbohydrate diet”
	Grade III: Severe inflammation–marked redness and oedema which covers ¾ or more of the crown of the affected tooth surface. Ulceration. Tendency to spontaneously bleed [33]	Studies focussing on children <18 years) were excluded
	Histopathological presentation:	
	1 Initial lesions	
	2 Early lesions	
	3 Established lesions [34]	
	(ii) Periodontal disease	
	Clinical presentation:	
	(i) “Attachment loss” of gingival tissues to the exterior surfaces of the tooth	
	(ii) Presence of gingival recession	
	(iii) Deep probing depths (more than >4 mm)	
	(iv) Furcation involvement	
	(v) Redness and oedema of surrounding gingival tissue	
	(vi) Bleeding on probing	
	Radiographic presentation	
	(i) Horizontal/vertical bone loss (lamina dura layer of the cortical bone)	
	Histopathological presentation:	
	(i) Transition from the established stage to the advanced stage [34]	
	(ii) Spread of inflammation from epithelium to connective tissue both apically and laterally with concomitant collagen fiber destruction [34]	

Study designs	RCTs, prospective cohort studies, retrospective case studies, retrospective cohort studies, descriptive studies, comparative studies, crossover studies, literature reviews, clinical trials, original research articles b	Abstracts, editorials, directories, nonEnglish articles, retracted studies, lectures, biographies, reviews, case reports, and other studies without a comparison group, cross-sectional studies, reports from conferences or annual meetings, editorials, opinions and in vitro studies were excluded from this review
Additional eligibility criteria for inclusion:	Articles stating relative risks (RRs), HRs or odds ratios (ORs) and corresponding 95% CIs, or data for their calculation a	
	Adult females with at least 10 teeth within the oral cavity, and with at least one tooth with a probing depth and intraorally determined gingival clinical attachment level of (CAL) ≥4 mm	

**Table 2:** Overall data from AXIS tool conducted studies *∗*. Calculated relative risk (RR), 95% CI and *P* values investigating the efficacy of FN from the oral microbiome and its biomarker potential for breast cancer in adult women.

Type of analysis	No. of studies	Heterogeneity		Results		
		*P*	*I* ^2^(%)	RR	95% CI	*P* value
Overall analysis (articles included from AXIS tool analysis only)	6	0.03	50.89	1.28	1.03–1.45	<0.01
Study design						
Prospective cohort	4	0.08	43.24	1.18	0.95–1.40	0.07
Retrospective cohort	1	0.35	40.65	1.22	1.11–1.39	<0.01
Case-control	1	—	—	2.10	1.15–2.78	0.04
Follow-up period^*∗∗*^						
<10 years	3	0.55	0.73	1.18	1.15–1.29	<0.01
≥10 years	2	0.05	65.94	1.41	0.88–1.87	0.06
Dental status						
PD	6	0.04	51.74	1.24	1.01–1.30	0.04

^*∗*^CASP-Tool examined articles were omitted due to insufficient statistical data. ^*∗∗*^ 1 Article omitted due to absence of follow-up period.

## Data Availability

All data have been provided in this article (main text). Additional data are accessible in the supplementary material section which can be accessed online.

## Abbreviations

FN: Fusobacterium nucleatum
BC: Breast cancer
PD: Periodontal disease
NHANES: National health and examination study
DHCPs: Dental health care professionals
ET 1: Endothelin 1
BDCIS: Breast ductal carcinoma *In Situ*
RCT: Randomised clinical trial
PST: Periodontal surgical trauma
SFA: Single flap approach
DFA: Double-flap approach
MIST: Minimally invasive surgical technique.

## Additional Points

Deeper understanding of how oral microbial imbalances potentiate/initiate/accelerate breast cancer pathogenesis in women would encourage more active discussions and collaborations between scientists, medical, and dental professionals. The adoption of a fabricated collaborative personalised patient care ethos would be revolutionary in its creation of a more structured, informed, and supportive environment for both patients and their treating clinician(s)-potentially saving many lives. This article (1) critically analyses evidence regarding the relationship between oral F.N and female-specific BC pathology. (2) Examines the link between endothelin (ET-1) molecules and oral FN species and their roles in inflammatory cytokine activation, PD and BC pathogenesis. (3) Discusses surgical treatments for PD (specifically periodontal flap designs) and the associated increase in risk of PD onset. (4) Illustrates the potentiality of oral FN species as biomarkers for BC pathology. (5) Compares the strengths and weaknesses of the presented evidence. (6) Raises awareness of the complex interactions of oral bacteria and inflammatory cytokines at a cellular level as a novel approach for therapeutic targets, thus improving future PD and BC prognosis.

## Supplementary Materials

Supplementary Materials Table 1: P.I.C.O.S point system criteria created for the purpose of article incorporation. Maximal score = 63. Table 2: P.I.C.O.S summary of combined results of articles *∗*-Best overall score of 63. Table 3: An illustration of MeSH terminology & complete search string utilised for the purposes of this systematic review. Figure 1. I: AXIS tool for articles investigating the link between Fusobacterium nucleatum from the oral micobiome and breast cancer patogenesis in premonopausal adult women: Tabular Representation II: AXIS tool for articles investigating the link between Fusobacterium nucleatum from the oral microbiome and breast cancer pathogenesis in premenopausal adult women–graphical representation. Description 1: A detailed description of the exact criteria originating from the EXPANDED PRISMA CHECKLIST 2020, utilised for the creation of this systematic review.
