# Supplementary material for: The Role of Oral Fusobacterium nucleatum in Female Breast Cancer: A Systematic Review and Meta-Analysis
Source: Int J Dent. 2022 Nov 23;2022:1876275. doi: 10.1155/2022/1876275 (PMC9711985; doi:10.1155/2022/1876275)
Supplement: Supplementary Materials — Table 1: P.I.C.O.S point system criteria created for the purpose of article incorporation. Maximal score = 63. Table 2: P.I.C.O.S summary of combined results of articles ∗-Best overall score of 63. Table 3: An illustration of MeSH terminology & complete search string utilised for the purposes of this systematic review. Figure 1. I: AXIS tool for articles investigating the link between Fusobacterium nucleatum from the oral micobiome and breast cancer patogenesis in premonopausal adult women: Tabular Representation II: AXIS tool for articles investigating the link between Fusobacterium nucleatum from the oral microbiome and breast cancer pathogenesis in premenopausal adult women–graphical representation. Description 1: A detailed description of the exact criteria originating from the EXPANDED PRISMA CHECKLIST 2020, utilised for the creation of this systematic review. [file 1876275.f1.docx]

**SUPPLEMENTARY APPENDICES – FIGURES, TABLES & DESCRIPTION(S).**

**
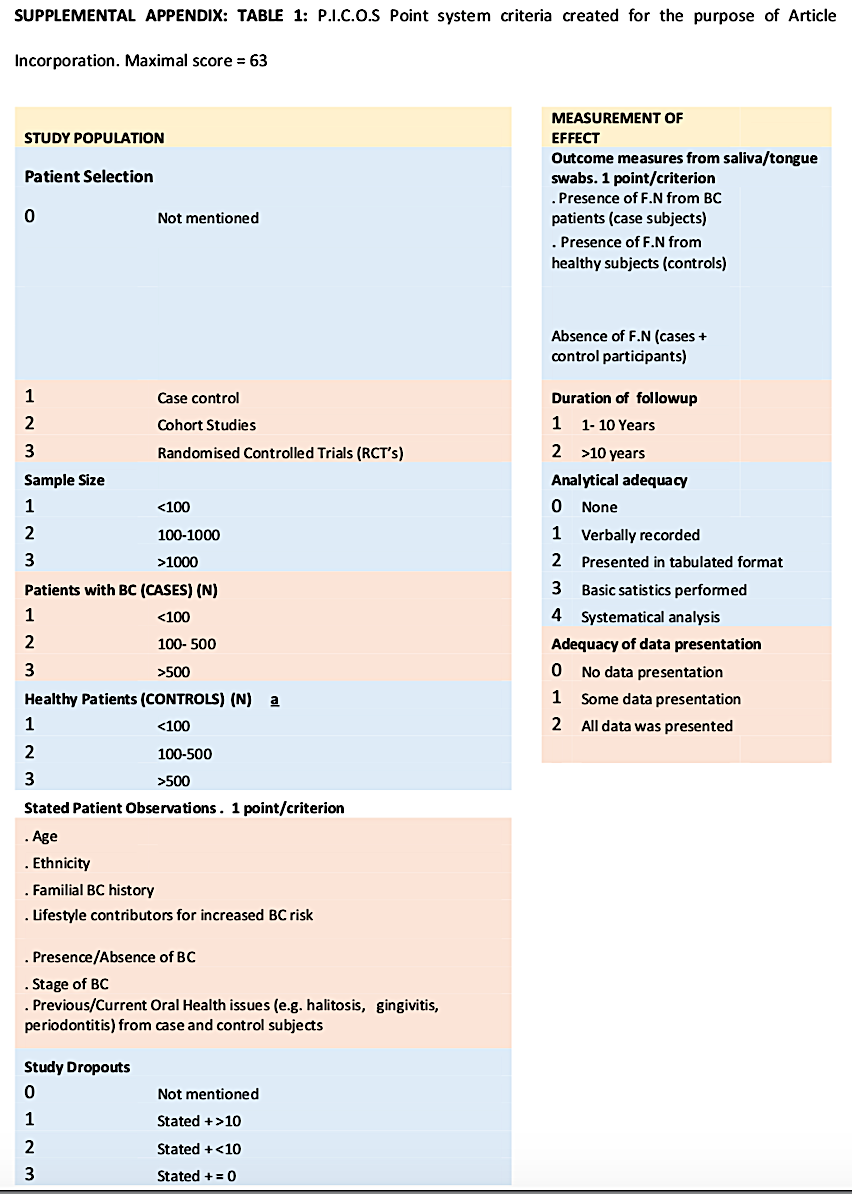
**

**
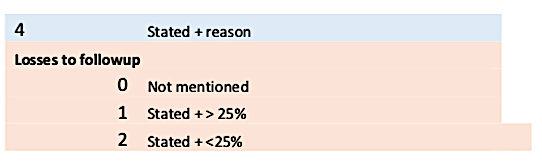
**

**SUPPLEMENTAL APPENDIX - TABLE 2: P.I.C.O.S Summary of combined results of Articles* - Best overall score of 63**

**
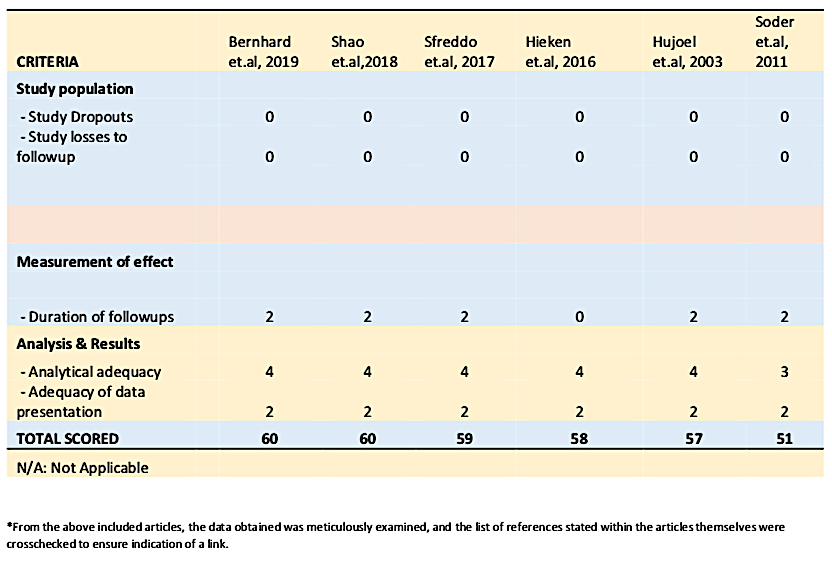
**

**SUPPLEMENTAL APPENDIX TABLE 3:** An illustration of MeSH Terminology & Complete search string utilised for the purposes of this Systematic Review.

| ***MeSH terms*** | ***OPTIONS*** | ***All Fields*** | ***OPTION***  ***S*** | ***All Fields*** | ***OPTION*** | ***All Fields*** |
| --- | --- | --- | --- | --- | --- | --- |
| mouth | **OR** | mouth | **OR** | oral | **AND** | **-** |
| Fusobacterium Nucleatum | **OR** | Fusobacterium | **AND** | Nucleatum | **OR** | Fusobacterium Nucleatum |
| - | **AND** | Breast  Neoplasms | **OR** | Breast | **AND** | Neoplasms |
| - | **OR** | Breast  Neoplasms | **OR** | Breast | **AND** | Cancer |
| - | **OR** | Breast Cancer | **AND** | Etiology | **OR** | Etiology |
| - | **OR** | Pathogenesis | **AND** | Adult | **OR** | Adult |
| - | **AND** | Women | **OR** | Women | **OR** | Biomarkers |
| Inflammatory  Mediators | **OR** | Inflammatory | **AND** | Mediators | **-** | - |
| Endothelins | **AND** | Endothelins | **-** | - | **-** | - |
| Interleukins | **AND** | IL-1, IL-6 | **OR** | IL-1 | **AND** | IL-6 |
|  | **AND** | IL-1, IL-8 | **OR** | IL-1 | **AND** | IL-8 |
| Fusobacterium Nucleatum  Analysis | **OR** | Fusobacterium Nucleatum | **AND** | Analysis | **OR** | Fusobacterium Nucleatum |
|  | **AND** | Metagenomics | **OR** | Fusobacterium Nucleatum | **AND** | Meta  Transcriptomics |
| Periodontitis | **AND** | Gum  inflammation | **AND** | Periodontitis | **AND** | Bone Loss |
|  | **OR** | Periodontitis | **AND** | Fusobacterium Nucleatum | **OR** | - |
|  | **-** | Periodontitis | **AND** | Surgery | **AND** | Flap Design |
|  | **OR** | Periodontitis | **AND** | Risk Factor | **AND** | Periodontal  Disease |
| Minimally  Invasive  Periodontal  Surgery with  Dental Loupes | **OR** | Minimally | **AND** | Invasive | **OR** | Minimally  Invasive |
|  | **OR** | Periodontal | **AND** | Surgery | **AND** | Dental Loupes |
|  | **OR** | Dental | **AND** | Loupes |  |  |

**Query Translation:**

**("mouth"[MeSH Terms] OR "mouth"[All Fields] OR "oral"[All Fields]) AND ("fusobacterium nucleatum"[MeSH Terms] OR ("fusobacterium"[All Fields] AND "nucleatum"[All Fields]) OR "fusobacterium nucleatum"[All Fields]) AND ("breast neoplasms"[MeSH Terms] OR ("breast"[All Fields] AND "neoplasms"[All Fields]) OR "breast neoplasms"[All Fields] OR ("breast"[All Fields] AND "cancer"[All Fields]) OR "breast cancer"[All Fields]) AND ("etiology"[Subheading] OR "etiology"[All Fields] OR "pathogenesis"[All Fields]) AND ("adult"[MeSH Terms] OR "adult"[All Fields]) AND ("women"[MeSH Terms] OR "women"[All Fields] OR “Biomarkers” [All Fields]. “Inflammatory Mediators”[MeSH Terms] OR (“Inflammatory” [All Fields]) AND (“Mediators” [All Fields]). “Edothelins” [MeSH Terms] AND “Endothelins”[All Fields]. “Interleukins” [MeSH Terms] AND (“IL-1 IL-6"[All Fields]) OR (“IL-1” [All Fields]) AND (“IL-6” [All Fields]) AND (“IL-1 IL-8"[All Fields]) OR (“IL-1” [All Fields]) AND (“IL-8” [All Fields]). “Fusobacterium Nucleatum Analysis” [MeSH Terms] OR (“Fusobacterium Nucleatum” [All Fields]) AND (“Analysis” [All Fields]) OR (“Fusobacterium Nucleatum”[All Fields]) AND (“Metagenomics” [All Fields]) OR (“Fusobacterium Nucleatum”[All Fields]) AND (“Meta-Transcriptomics” [All Fields]). “Periodontitis” [MeSH Terms] AND (“Gum Inflammation” [All Fields] AND “Periodontitis”[All Fields] AND “Bone Loss” [All Fileds] ) OR (“Periodontitis” [All Fields]) AND (“Fusobacterium Nucleatum”[all Fields]) OR (“Periodontitis”[All Fields] AND (“Surgery”[All Fields]) AND (“Flap-Design”[All Fields]) OR (“Periodontitis”[All Fields]) AND (“Risk Factor” [All Fields]) AND (“ Periodontal Disease”[All Fields]). “Minimally Invasive Periodontal Surgery with Dental Loupes” [MeSH Terms] OR (“Minimally” [All Fields]) AND (“Invasive” [All Fields]) OR (“Minimally Invasive” [All Fields] OR (“Periodontal” [All Fields]) AND “Surgery” [All Fields]) AND (“Dental Loupes” [All Fields]) OR (“Dental” [All Fields]) AND (“Loupes” [All Fields])**

**SUPPLEMENTAL APPENDIX FIGURE 1I:** AXIS Tool for Articles Investigating the link between Fusobacterium Nucleatum from the Oral Microbiome and Breast Cancer Pathogenesis in Premenopausal Adult Women: Tabular Representation
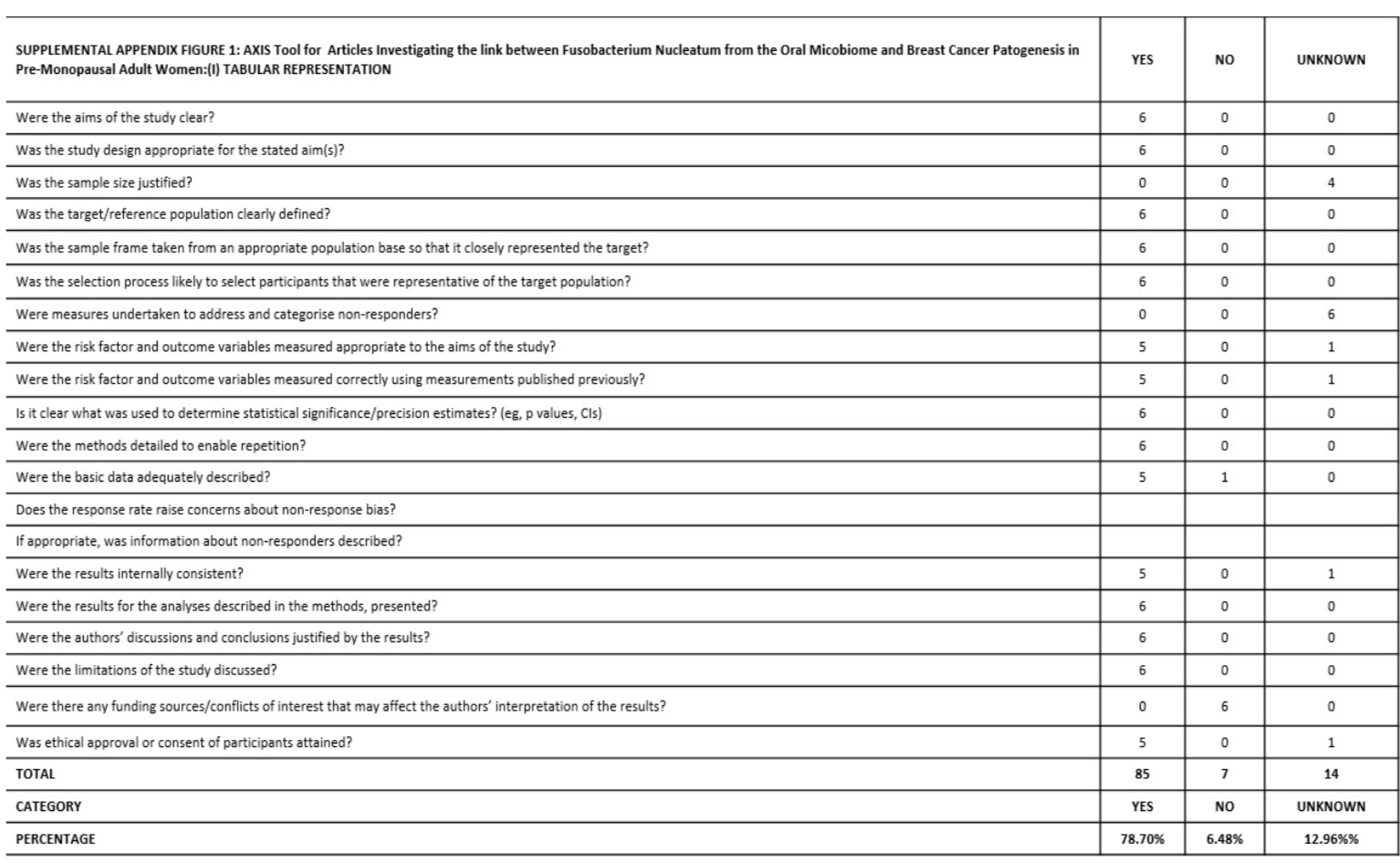


**FIGURE 1II**– AXIS Tool for Articles investigating the link between Fusobacterium Nucleatum from the Oral Microbiome and Breast Cancer Pathogenesis in Pre-Menopausal Adult Women – Graphical Representation


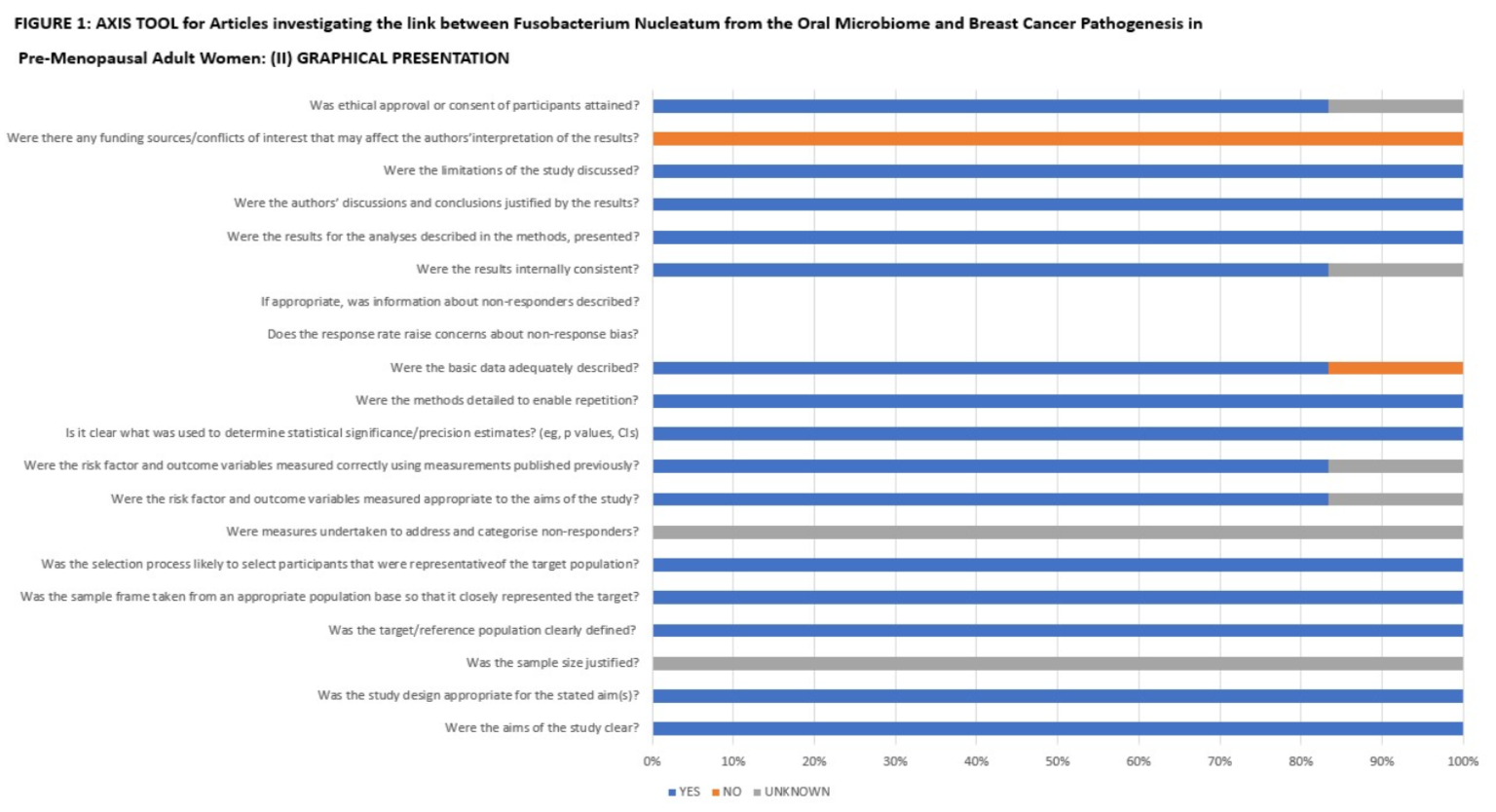


**DESCRIPTION 1:** A detailed description of the exact criteria originating from the **EXPANDED PRISMA CHECKLIST 2020,** utilised for the creation of this Systematic Review

**TITLE**

1 • Identify the report as a systematic review in the title.

• Reportation of an informative title that provides key information about the main objective or question the review addresses (e.g. the population(s) and intervention(s) the review addresses)

**ABSTRACT**

2 • Reportation of an abstract addressing each item in the PRISMA 2020 for Abstracts checklist.

**INTRODUCTION**

**RATIONALE**

3 • Description of the current state of knowledge and its uncertainties.

• Articulation of why it is important to do the review - declared.

OBJECTIVES

4 • Statement of all objective(s) or question(s) the review addresses, expressed in terms of a relevant question formulation framework.

• If the purpose is to evaluate the effects of interventions, use the Population, Intervention, Comparator, Outcome (PICO) framework or one of its variants, to state the comparisons that will be made - declared.

**METHODS**

**ELIGIBILITY CRITERIA**

5 • Specify all study characteristics used to decide whether a study was eligible for inclusion in the review, that is, components described in the PICO framework or one of its variants, and other characteristics, such as eligible study design(s) and setting(s), and minimum duration of follow-up. - Done.

• Specify eligibility criteria with regard to report characteristics, such as year of dissemination, language, and report status (e.g. whether reports, such as unpublished manuscripts and conference abstracts, were eligible for inclusion). Done

• Clearly indicate if studies were ineligible because the outcomes of interest were not measured, or ineligible because the results for the outcome of interest were not reported. Done

• Specify any groups used in the synthesis (e.g. intervention, outcome and population groups) and link these to the comparisons specified in the objectives (item #4). Described.

**SOURCES**

6 • Specify the date when each source (e.g. database, register, website, organisation) was last searched or consulted. Specfied.

• If bibliographic databases were searched, specify for each database its name (e.g. MEDLINE, CINAHL), the interface or platform through which the database was searched (e.g. Ovid, EBSCOhost), and the dates of coverage (where this information is provided). Declared.

**SEARCH STRATEGY**

7 • Provide the full line by line search strategy as run in each database with a sophisticated interface (such as Ovid), or the sequence of terms that were used to search simpler interfaces, such as search engines or websites. Done

• Describe any limits applied to the search strategy (e.g. date or language) and justify these by linking back to the review’s eligibility criteria. Declared

**SELECTION PROCESS**

8 Recommendations for reporting regardless of the selection processes used:

• Report how many reviewers screened each record (title/abstract) and each report retrieved, whether multiple reviewers worked independently at each stage of screening or not, and any processes used to resolve disagreements between screeners. Described.

• Report any processes used to obtain or confirm relevant information from study investigators. Reported.

**DATA COLLECTION**

**PROCESS**

9 • Report how many reviewers collected data , whether multiple reviewers worked independently or not, and any processes used to resolve disagreements between data collectors. Reported

• Report any processes used to obtain or confirm relevant data from study investigators. Described.

**DATA ITEMS (outcomes)**

10a • List and define the outcome domains and time frame of measurement for which data were sought. Described.

**STUDY RISK OF BIAS**

**ASSESSMENT**

11• Specify the methodological domains/components/items of the risk of bias tool(s) used. Done.

• Report whether an overall risk of bias judgement that summarised across domains/components/items was made, and if so, what rules were used to reach an overall judgement. Described.

**EFFECT MEASURES**

12 • Specify for each outcome (or type of outcome [e.g. binary, continuous]), the effect measure(s) (e.g. risk ratio, mean difference) used in the synthesis or presentation of results. Specified.

**SYNTHESIS METHODS**

(eligibility for synthesis)

13a • Describe the processes used to decide which studies were eligible for each synthesis. Completed.

**SYNTHESIS METHODS**

(tabulation and graphical methods)

• Report chosen tabular structure(s) used to display results of individual studies and syntheses, along with details of the data presented. Reported

• Report chosen graphical methods used to visually display results of individual studies and syntheses. Reported

**SYNTHESIS METHODS**

(statistical synthesis methods)

13d •If meta-analysis was done, specify: any methods used to identify or quantify statistical heterogeneity (e.g. visual inspection of results, a formal statistical test for heterogeneity, heterogeneity variance (��2), inconsistency (e.g. I2), and prediction intervals). Specified.

**REPORTING BIAS ASSESSMENT**

14 • Report how many reviewers assessed risk of bias due to missing results in a synthesis, whether multiple reviewers worked independently, and any processes used to resolve disagreements between assessors. Reported.

• Report any processes used to obtain or confirm relevant information from study investigators. Described.

**CERTAINTY ASSESSMENT**

15. Report the factors considered (e.g. precision of the effect estimate, consistency of findings across studies) and the criteria used to assess each factor when assessing certainty in the body of evidence. Reported.

**RESULTS OF SYNTHESES**

(results of statistical syntheses)

20b • If meta-analysis was conducted, report for each:

o the summary estimate and its precision (e.g. standard error or 95% confidence/credible interval). Reported o measures of statistical heterogeneity (e.g. ��2, I2, prediction interval). Reported

20c• If subgroup analysis was conducted:

o report for each analysis the exact P value for a test for interaction, as well as, within each subgroup, the summary estimates, their precision (e.g. standard error or 95% confidence/credible interval) and measures of heterogeneity.) Reported.

**DISCUSSION**

(interpretation)

23a • Provide a general interpretation of the results in the context of other evidence. Provided

**DISCUSSION**

(limitations of evidence)

23b • Discuss any limitations of the evidence included in the review. Discussed.

**DISCUSSION**

(limitations of review processes)

23c • Discuss any limitations of the review processes used, and comment on the potential impact of each limitation. Discussed

**DISCUSSION**

(implications)

23d • Discuss implications of the results for practice and policy. Make explicit recommendations for future research. Discussed

**COMPETING INTERESTS**

26 • Disclose any of the authors’ relationships or activities that readers could consider pertinent or to have influenced the review.

• If any authors had competing interests, report how they were managed for particular review processes. Disclosed.

**AVAILABILITY OF DATA, CODE, AND OTHER MATERIALS**

27 • Report which of the following are publicly available: template data collection forms; data extracted from included studies; data used for all analyses; analytic code; any other materials used in the review. Data availability statement in main article present.
